# Growth and development of children under 5 years of age with tetralogy of Fallot in a Chinese population

**DOI:** 10.1038/s41598-021-93726-3

**Published:** 2021-07-09

**Authors:** Xin Li, Jin Zhu, Jun An, Yuqing Wang, Yili Wu, Xuezhi Li

**Affiliations:** 1 Department of Pediatrics, TEDA International Cardiovascular Hospital, TEDA, Tianjin, China; 2 Department of Pediatric Cardiac Surgery, TEDA International Cardiovascular Hospital, TEDA, Tianjin, China; 3 Shandong Collaborative Innovation Center for Diagnosis, Treatment and Behavioral Interventions of Mental Disorders, Institute of Mental Health, Jining Medical University, Jining, China; 4 Shandong Key Laboratory of Behavioral Medicine, School of Mental Health, Jining Medical University, Jining, China; 5 Department of Biochemistry and Molecular Biology, College of Life Sciences, Nankai University, Tianjin, China; 6 Key Laboratory of Alzheimer’s Disease of Zhejiang Province, Institute of Aging, Wenzhou Medical University, Wenzhou, Zhejiang China; 7 Oujiang Laboratory, Wenzhou, Zhejiang China

**Keywords:** Congenital heart defects, Malnutrition

## Abstract

Congenital Heart Defects (CHDs) are associated with different patterns of malnutrition and growth retardation, which may vary worldwide and need to be evaluated according to local conditions. Although tetralogy of Fallot (TOF) is one of the first described CHDs, the etiology outcomes in growth and development of TOF in early age child is still unclear in most cases. This study was designed to investigate the growth retardation status of Chinese pediatric TOF patients under 5 years old. The body height, body weight and body mass index (BMI) of 262 pediatric patients (138 boys and 124 girls) who underwent corrective surgery for TOF between 2014 and 2018 were measured using conventional methods. The average body height, body weight and BMI of the patients were significantly lower than *WHO Child Growth Standards*, while the most affected was body height. Meanwhile, higher stunting frequency and greater deterioration of both the body height and weight happened in elder age (aged 13–60 months) rather than in infant stage (aged 0–12 months) among these patients. Our results confirmed that intervention should be given at early age to prevent the growth retardation of TOF patients getting severer.

## Introduction

Tetralogy of Fallot (TOF) is one of the most common Congenital Heart Defects (CHDs), named for French physician Dr. Etienne-Louis Arthur Fallot, who described a panel of three cyanotic patients in 1888 with four anatomical features: (1) stenosis of the pulmonary artery, (2) interventricular communication (VSD), (3) deviation of the origin of the aorta (overriding aorta), and (4) concentric right ventricular hypertrophy^1–4^. Based on a meta-analysis in 2011, TOF is the fourth prevailing CHD type worldwide with a prevalence of 0.34‰, constituting approximately 4% of CHD cases overall^5^. Situation of TOF in China differs. According to the national wide epidemiological survey of CHD in China directed by Hubei Women and Child Healthcare Hospital between 2015 and 2017, TOF is the most prevailing CHD type in new born children in China^6^.

Children with a range of chronic systemic health conditions often has significant adverse effects on body growth and development^7^. Indeed, different CHD types are associated with different patterns of malnutrition and growth retardation, which may be caused by prenatal and genetic factors, hypoxia and hemodynamic factors, and those relating to nutritional intake, metabolic requirements, and nutrient absorption^7–12^. As some of these factors may vary worldwide, CHD’s effects on growth and development needs to be evaluated according to local conditions. The impact of CHD on growth and development has been almost eliminated in benefit of modern medical advantages and well concern on patients in developed countries^8^. But most of the CHD cases are diagnosed and treated late due to poor resource availability in developing countries^9^. The understanding and prevention of the effect of CHD on patients’ growth and development status in developing countries still needs improvement. Although TOF is the most prevalent fetal CHD type in China, its outcomes on pediatric growth and development are not well investigated. This study was designed to specially evaluate the growth and development of children under 5 years old with TOF in China.

## Results

**Figure 1:**
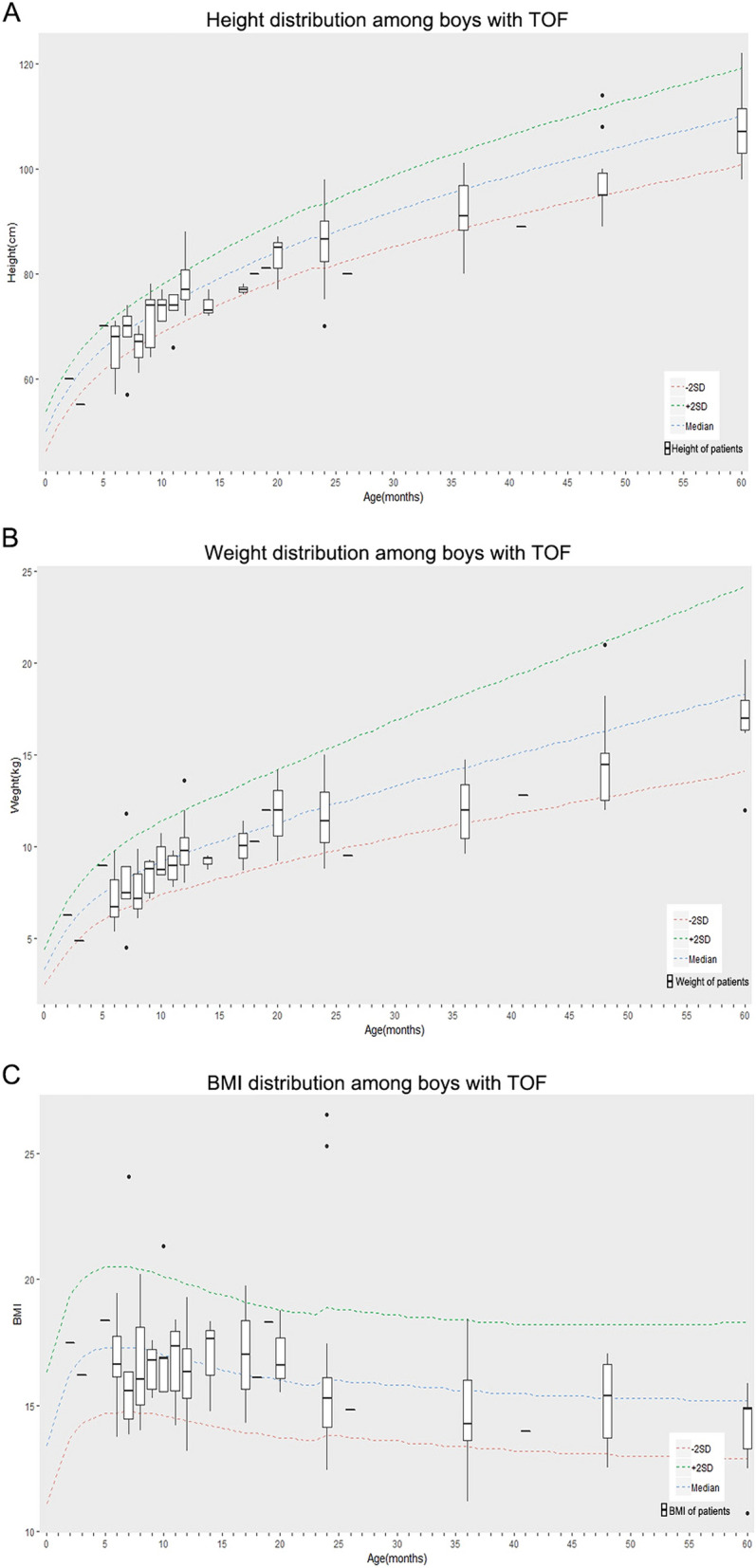
Growth charts for boys with TOF (total number = 138). (**A**) Height for age in TOF patient boys compared with normal chart. (**B**) Weight for age in TOF patient boys compared with normal chart. (**C**) BMI for age in TOF patient boys compared with normal chart. The dotted line represents the world standard value for the height, the green dotted line represents + 2SD, the blue dotted line represents Median, and the red represents -2SD. The data are calculated based on gender and age and calculated in the R Programming Language.

**Figure 2:**
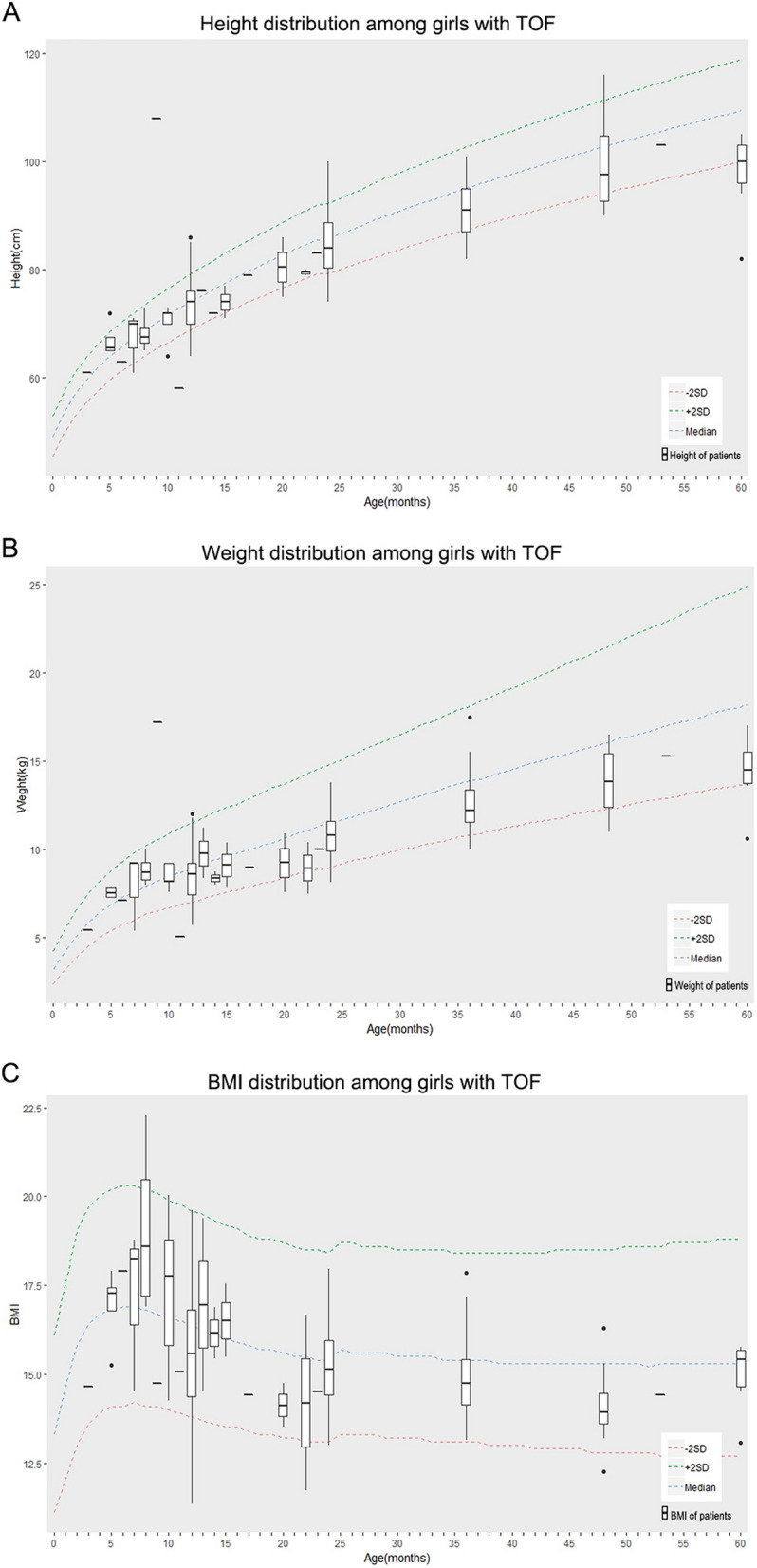
Growth charts for girls with TOF (total number = 124). (**A**) Height for age in TOF patient girls compared with normal chart. (**B**) Weight for age in TOF patient girls compared with normal chart. (**C**) BMI for age in TOF patient girls compared with normal chart. The dotted line represents the world standard value for the height, the green dotted line represents + 2SD, the blue dotted line represents Median, and the red represents -2SD. The data are calculated based on gender and age and calculated in the R Programming Language.

The age information, mean *z* score and standard deviation (SD) of height, weight and BMI of all the 262 pediatric patients included in this study are summarized in Table 1 and detailed shown in Table S1. Growth charts of girls and boys with TOF are shown in Figs. 1 and 2 independently.

**Table 1:** The height, weight and BMI Z-scores of patients with TOF.

Retardation pattern	Girls				Boys			
	Total (n = 124)	Infant (n = 49)	Child (n = 75)	*P* value	Total (n = 138)	Infant (n = 65)	Child (n = 73)	*P* value
HAZ	− 0.44 ± 2.40	0.22 ± 3.14	− 0.87 ± 1.67	0.176	− 0.34 ± 2.07	0.25 ± 2.20	− 0.88 ± 1.80	0.551
WAZ	− 0.52 ± 1.73	0.03 ± 2.25	− 0.88 ± 1.16	0.22	− 0.44 ± 1.52	− 0.04 ± 1.62	− 0.80 ± 1.33	0.612
BMIZ	− 0.37 ± 1.32	− 0.31 ± 1.60	− 0.41 ± 1.11	0.379	− 0.31 ± 1.88	− 0.33 ± 1.53	− 0.29 ± 2.16	0.04*

z scores are shown as mean ± SD. Z scores of individuals from infant group and child group were compared using paired samples *t* test. *P < 0.05 was considered as significant difference.

### Growth and development status of children with TOF

The patients’ body height, body weight and BMI were compared to the *WHO Child Growth Standards* released in 2006. In order to evaluate the age specific growth and development status, the patients were set to two groups including infant patients (aged 0–12 months) and child patients (aged 13–60 months). The data of girls and boys were analyzed independently (Table 1). For the girl patients, the average z scores of body height, body weight and BMI were about 0.44, 0.52 and 0.37 less than the value from Growth Standards, respectively. The differences of average z-scores of body height, body weight and BMI were greater in child groups than infant groups (− 0.87 vs. − 0.22 for HAZ, *P* = 0.176; − 0.88 vs. 0.03 for WAZ, *P* = 0.22; − 0.41 vs. − 0.31 for BMIZ, *P* = 0.379). For the boy patients, the average z-scores of body height, body weight and BMI were about 0.44, 0.34 and 0.31 less than the value from Growth Standards, respectively. Moreover, the differences of average z scores of body height and body weight were greater in child groups than infant groups (− 0.80 vs. − 0.04 for HAZ, *P* = 0.551; − 0.88 vs. − 0.25 for WAZ, *P* = 0.612). The differences of average BMIZ didn’t show big variation between child groups and infant groups (− 0.29 vs. − 0.0.33, *P* = 0.04).

### Growth retardation patterns of the pediatric TOF patients

To further define the effects of TOF on growth and development, stunting (low HAZ), underweight (low WAZ) and wasting (low BMIZ) frequencies were evaluated. A cut-off *z* score of ≤ − 2 was used to classify stunting, underweight and wasting, while a cut off z score of ≤ − 3 was classified as severe stunting, severe underweight and severe wasting^8,10^. The stunting, underweight and wasting frequencies of girl patients and boy patients are shown in Table 2.

**Table 2:** The growth retardation frequencies of patients with TOF.

Retardation pattern	Girls				Boys				Total			
	Total (n = 124)	Infant (n = 49)	Child (n = 75)	*P* value	Total (n = 138)	Infant (n = 65)	Child (n = 73)	*P* value	Total (n = 262)	Infant (n = 114)	Child (n = 148)	*P* value
Stunting (HAZ ≤ − 2)	29 (23.4%)	9 (18.4%)	20 (26.7%)	0.286	27 (19.6%)	8 (12.3%)	19 (26.0%)	0.011*	56 (21.4%)	17 (14.9%)	39 (26.4%)	0.025*
Severe stunting (HAZ ≤ − 3)	6 (4.8%)	2 (4.1%)	4 (5.3%)	1.000	13 (9.4%)	4 (6.2%)	6 (8.2%)	0.749	16 (6.1%)	6 (5.3%)	10 (6.8%)	0.617
Underweight (WAZ ≤ − 2)	18 (14.5%)	7 (14.3%)	11 (14.7%)	0.953	20 (14.5%)	5 (7.7%)	15 (20.5%)	0.032*	38 (14.5%)	12 (10.5%)	26 (17.6%)	0.109
Severe underweight (WAZ ≤ − 3)	7 (5.6%)	5 (10.2%)	2 (2.7%)	0.112	6 (4.3%)	3 (4.6%)	3 (4.1%)	0.666	13 (5.0%)	8 (7.0%)	5 (3.4%)	0.098
Wasting (BMIZ ≤ − 2)	8 (6.5%)	5 (10.2%)	3 (4.0%)	0.262	18 (13.0%)	9 (13.8%)	9 (12.3%)	0.792	26 (9.9%)	14 (12.3%)	12 (8.1%)	0.263
Severe wasting (BMIZ ≤ − 3)	5 (4.0%)	4 (8.2%)	1 (1.3%)	0.079	6 (4.3%)	2 (3.1%)	4 (5.5%)	0.684	11 (4.2%)	6 (5.3%)	5 (3.4%)	0.540

Wasting frequency of infant group and child group were compared using chi-square test. *P* < 0.05 was considered as significant difference.

Of the 262 patients, 56 cases (21.4%) were stunting, including 16 cases (6.1%) were severe stunting (Table 2). Underweight frequency was lower than the frequency of stunting, 38 (14.5%) patients were stunting with 12 (4.6%) severe cases (Table 2). 26 cases (9.9%) were wasting, including 11 severe wasting cases (4.2%) (Table 2). Stunting was the most occurred growth retardation pattern both in girls (n = 29, 23.4%) and boys (n = 27, 19.6%), while severe stunting show the highest frequency in boys (n = 13, 9.4%).

**Figure 3:**
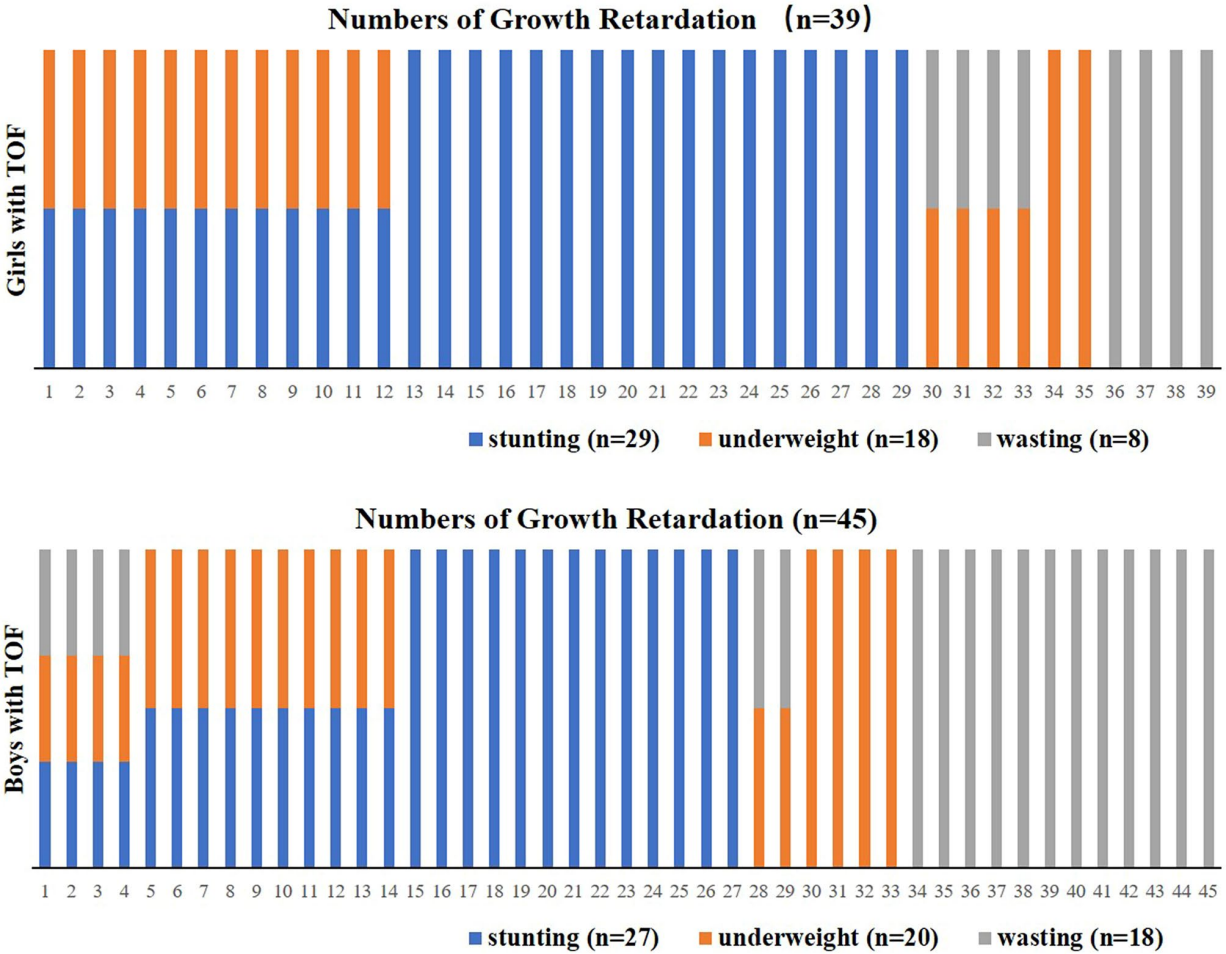
Schematic summary of growth retardation pattern distribution in TOF patients. The number of each growth retardation pattern in the 124 girls and 138 boys with TOF were calculated based on the original data and summarized.

The detailed distribution of stunting, underweight and wasting patient numbers are summarized in Fig. 3. There were totally 39 girl patients (31.5% of the 124 girl patients) had growth retardation, including 12 patients (9.7%) had both stunting and underweight, 4 patients (3.2%) had both underweight and wasting. Of the 138 boy patients, 45 cases had growth retardation, including 4 cases (2.9%) had all the three patterns, 10 cases (7.2%) had both stunting and underweight, and 2 cases (1.4%) had both underweight and wasting.

As shown in Table 2, there was an increasing trend of stunting in child groups of both in girl (26.7% in child vs. 18.7% in infant, *P* = 0.286) and boy patients (26.0% in child vs. 12.3% in infant, *P* = 0.011). The underweight frequency of child group of boy patients was significantly increased compared with infant group (20.5% in child vs. 7.7% in infant, *P* = 0.032). Interestingly, the wasting frequencies exhibited a decrease trend in child group compared with infant group (10.2% in child vs. 4.0% in infant, *P* = 0.262) of girl patients.

## Discussion

Increasing evidences suggest that different types of cardiac defects are associated with different patterns of growth retardation, while the situation also varies among nations^11–13^. Our study specially evaluated the growth and development of children with TOF, the most prevailing CHD type in China.

A survey in United States reported in 1962 showed that 27% of the children with CHD would fall below the third percentile (equal to a z score of − 1.881) for height and weight^14^. But updated researches indicated that the impact of CHD on growth and development has been almost eliminated in developed countries, but still needed more concern in other counties^8,9^. The recent study in Egypt showed the relative proportions of stunting, underweight and wasting of CHD patients were 61.9%, 14.3% and 23.8%, respectively^15^. Okoromah et al. reported in 2011 that the relative proportions of stunting, underweight and wasting (z score ≤ − 2, defined as malnutrition) of surveyed CHD children in Nigeria were 28.8%, 20.5%, and 41.1%, respectively^8^. For Asia countries, a study in 2011 reported that 22.6% of CHD patients were below the fifth percentile (equal to a z-score of − 1.645) for height, while 40.3% of patients were below the fifth percentile for weight in Iran^10^. In the same year, Ratanachu et al*.* revealed that relative proportions of stunting, underweight and wasting in Thailand children with CHD were 16%, 28% and 22%, respectively^16^. In our study, the relative proportions of stunting, underweight and wasting of children with TOF in our study were 21.4%, 14.5% and 9.9%, respectively. Our findings were somehow lower than those recent reports from other countries.

Despite that the other reports were about CHD and we specifically focused on TOF, the reason of the differences in the proportion and the pattern of growth retardation among each independent studies possibly due to different cut-off *z* scores, CHD type and Nations, which are effectors mentioned in previous reports^8,17,18^. Hassan, B. A. et al. reported that stunting was the most common type of malnutrition and was more linked to acyanotic CHD, while wasting was more associated with cyanotic CHD (~ 50% patients with TOF) in their study^15^. In contrast, Okoromah et al. reported that children with cyanotic CHD (~ 44% patients with TOF) were more likely to be stunting^8^. However, they haven’t distinguished the malnutrition pattern of children with TOF separately. In our study, body height was more affected in children with TOF. It has been reported that TOF affects body height severer than acyanotic CHD, might be due to the long period of hypoxemia^19^. The four defects caused by TOF results in short of blood in lung to get oxygen, and oxygen-poor blood effects the metabolic in body tissues, finally leads to the defects of growth and development.

Although it is generally thought that catch-up growth is better in patients operated at early age, FM Schuurmans et al*.*’s study found no significant relationship between the age when the child having surgical intervention and the catch-up growth^20^. They also reported that the most severe growth defection occurred in the 0–4 months old and turn to be slighter in elder age. In agreement with this report, Mohammad Dalili et al. found children older than 12 months had slighter defections than infant (0–12 months) ^10^. These reports suggested that growth retardation has already happened at early age (0–12 months) and will get slighter when grow up, which indicates early age is not a crucial time point for surgical intervention. In contrast, we found stunting frequency significantly raised from 14.9% in infant (aged 0–12 months) with TOF to 26.4% in children (aged 13–60 months) with TOF. Meanwhile, the underweight frequency raised from 10.5% to 17.6%, although is not significant. On the other hand, reduces of mean body height and body weight TOF patients from the standard value were amplified in children group (aged 13–60 months). Our results suggest greater deterioration of both the body height and weight of TOF patients happen in elder age rather than infant stage in China. Thus, early intervention for TOF patient may help to prevent the growth retardation getting severer. The average values of BMI and the frequencies of wasting didn’t show big variation between child and infant groups in our study. It is possibly due to some patients simultaneously have low body height (stunting) and low body weight (underweight).

It is reported that not only diseases, but also multiple factors, such as ethnicity, socioeconomic, even the education level of parents and the feeding behavior can effect growth and development status of children^21–24^. Thus, the cause and intervention for growth retardation may varied among different countries. However, for the growth retardation caused by CHD, surgical operation together with nutritional optimization are of the effective prevention strategy^25,26^. Based on our results, at least in the situation of China, intervention for patient with TOF, such as surgical operation and aggressive nutritional optimization should be considered at earlier age^27,28^.

## Methods

The study includes 262 pediatric patients aging from 1 month to 5 years who undergo corrective surgery for TOF at the department of pediatric cardiology, TEDA International Cardiovascular Hospital, Tianjin, China between 2014 and 2018. All the patients had not undergone palliative stenting or shunts or had undergone corrective surgery before coming to our hospital for treatment. The age information is shown in Table S1.

This study was carried out in accordance with the recommendations of clinical practice guidelines of China (Chinese Medical Association) with written informed consent from all subjects. Informed consent was obtained from the children’s guardian. The protocol was approved by the Ethical Committee of TEDA International Cardiovascular Hospital. All patients’ cardiac diagnoses were made based on clinical and laboratory examinations. Informed consent was obtained from the children’s guardian. A full medical history as well as a complete cardiac examination were documented for all children. Complete physical examination was performed for all patients by pediatric cardiologists and standardized measurements of body length、height and weight were done by trained nurses. Body mass index (BMI) was calculated by weight/height^2^.

*Z* scores for height for age (*HAZ*), weight for age (*WAZ*) and body mass index (*BMIZ*) were calculated using the Anthropometric calculator module of WHO Anthro software (based on *the WHO child growth standards*). Briefly, the *z* score refers to the ratio of the difference between the measured value and the standard median value recommended by the *WHO child growth standards (2006)*. The *z* score was calculated by the following formula: *z score* = (*real measured value*—*median value of the standards*)/*standard deviation of the WHO child growth standards.* Bigger *z* score indicates the value is farther from the median level. The three common indicators related to height and weight are *HAZ*, *WAZ*, *BMIZ*. In a series of growth curves, between plus and minus 2 standard deviations (> − 2SD and < 2SD) is considered as normal. According to the *Assessment for growth status of children under 5 years of age* issued by National Health Commission of the People’s Republic of China in 2013, *HAZ* ≤ − 2 is classified as stunting, *WAZ* ≤ − 2 is classified as underweight, *BMIZ* ≤ − 2 is classified as wasting. The WHO global database on child growth and malnutrition (under-nutrition) also recommends a cut-off *z* score of ≤ − 2 to classify low HAZ (stunting), low WAZ (underweight), and low BMIZ (wasting).

Statistical analysis: In each group, the data was analyzed based on gender. Body height and weight and of each group were compared with standard values for the same age group. Data were analyzed using the Statistical Package for Social Sciences (SPSS) release16 and presented as mean ± SD. A *P*-value of < 0.05 was considered statistically significant.

## Supplementary Information

Supplementary Information.
