# Supplementary material for: Growth and development of children under 5 years of age with tetralogy of Fallot in a Chinese population
Source: Sci Rep. 2021 Jul 9;11:14255. doi: 10.1038/s41598-021-93726-3 (PMC8271005; doi:10.1038/s41598-021-93726-3)
Supplement: Supplementary file 1 — Supplementary Information. [file 41598_2021_93726_MOESM1_ESM.pdf]

SUPPLEMENTARY Information SECTION

Growth and development of children under 5 years of age with tetralogy of Fallot in a Chinese population

Xin Li[1,2#*], Jin Zhu[3,4#], Jun An[5], Yuqing Wang[3,4], Yili Wu[3,4,6,7*], Xuezhi Li[3,4*]

[1] Department of Pediatrics, TEDA International Cardiovascular Hospital, TEDA, Tianjin, China
[2] Department of Pediatric Cardiac Surgery, TEDA International Cardiovascular Hospital, TEDA, Tianjin, China
[3] Shandong Collaborative Innovation Center for Diagnosis, Treatment and Behavioral Interventions of Mental Disorders, Institute of Mental Health, Jining Medical University, Jining, China
[4] Shandong Key Laboratory of Behavioral Medicine, School of Mental Health, Jining Medical University, Jining, China
[5] Department of Biochemistry and Molecular Biology, College of Life Sciences, Nankai University, Tianjin, China
[6] Key Laboratory of Alzheimer's Disease Of Zhejiang Province, Institute of Aging, Wenzhou Medical University, Wenzhou 325000, Zhejiang, China
[7] Oujiang Laboratory, Wenzhou 325000, Zhejiang, China

# The authors contribute equally to this study.
*Correspondence:
Xuezhi Li: lixuezhi@ mail.jnmc.edu.cn
Yili Wu: yili_wu2004@yahoo.ca; wuyili@mail.jnmc.edu.cn
Xin Li: an9pig@126.com

Table S1. The height, weight and BMI *Z*-scores according to age in girls with TOF

Age (month)		girls				boys			
		n	HAZ	WAZ	BMIZ	n	HAZ	WAZ	BMIZ
infant (0-12)	2	/				1	0.80	1.08	0.92
	3	1	0.57	-0.56	-1.25	1	-3.12	-2.14	-0.54
	5	4	1.36±1.53	0.90±0.43	0.10±0.85	1	1.91	2.00	0.82
	6	1	-1.2	-0.25	0.35	5	-0.93±2.76	-0.84±2.33	-0.43±1.61
	7	3	0.01±2.39	0.41±2.74	0.21±1.72	5	-0.45±3.02	-0.39±3.33	-0.35±3.32
	8	4	-0.19±1.45	1.19±1.09	1.69±1.86	3	-2.09±2.08	-1.02±2.30	-0.42±2.43
	9	1	15.79	10.59	-1.50	5	-0.27±2.70	-0.56±1.09	-0.56±0.80
	10	5	-0.52±1.45	-0.02±0.78	0.56±1.77	5	0.13±1.13	0.12±1.14	0.19±1.99
	11	1	-5.80	-4.03	-1.10	5	-0.65±1.79	-0.60±0.93	-0.17±1.46
	12	29	-0.03±2.27	-0.48±1.65	-0.77±1.46	34	1.00±1.93	0.33±1.29	-0.44±1.25
	total	49	0.22±3.14	0.03±2.25	-0.31±1.60	65	0.25±2.20	-0.04±1.62	-0.33±1.53
child (13-60)	13	2	0.31	0.59±1.99	0.60±2.76				
	14	2	-1.63	-1.03±0.53	0.04±0.82	3	-1.63±1.08	-0.88±0.41	0.25±1.58
	15	2	-1.27±1.54	-0.5±1.84	0.41±1.17				
	17	1	-0.25	-1.8	-1.15				
	18	/				1	-0.85	-0.57	-0.01
	19	/				1	-0.80	0.82	1.90
	20	2	-0.73±2.59	-1.23±1.12	-1.23±0.72	3	-0.43±1.89	0.45±2.28	0.84±1.44
	22	2	-1.65±0.23	-1.79±1.71	-1.09±1.71	/			
	23	1	-0.79	-1.08	-0.77	/			
	24	22	-0.36±1.95	-0.52±1.17	-0.20±1.17	28	-0.49±2.10	-0.58±1.31	-0.15±2.83
	26	/				1	-2.79	-2.40	-0.96
	36	23	-0.92±1.31	-0.88±1.11	-0.40±1.11	14	-1.15±1.73	-1.45±1.15	-0.84±1.77
	41	/				1	-2.62	-1.45	-1.34
	48	10	-0.77±1.91	-1.16±0.98	-0.98±0.90	12	-0.17±1.61	-0.39±1.49	-0.32±1.41
	53	1	-0.58	-0.83	-0.68	/			
	60	7	-2.43±1.67	-1.70±0.91	-0.24±0.75	7	-0.43±1.72	-0.69±1.20	-1.06±1.54
	total	75	-0.87±1.67	-0.88±1.16	-0.41±1.11	/			
total cases		124	-0.44±2.40	-0.52±1.73	-0.37±1.32	138	-0.34±2.07	-0.44±1.52	-0.31±1.88
